# Genetic characterization of a canine model of human GAPO syndrome caused by a nonsense variant in *ANTXR1*

**DOI:** 10.1371/journal.pone.0359081

**Published:** 2026-10-26

**Authors:** Katherine Stanbury, Maria Paola Cassarani, Joseph P. Boyle, Ellen Schofield, Bryan McLaughlin, Cathryn Mellersh

**Affiliations:** 1 Department of Veterinary Medicine, University of Cambridge, Cambridge, United Kingdom; 2 Centro Veterinario Oculistico Argo, Ancona, Italy; Astellas, UNITED STATES OF AMERICA

## Abstract

GAPO syndrome is characterised by a core combination of phenotypes: **g**rowth retardation, **a**lopecia, **p**seudoanodontia and **o**cular atrophy, and to date has only been reported in humans. It is a rare autosomal recessive condition caused by pathogenic variants in the Anthrax Toxin Receptor 1 (*ANTXR1*) gene. This study reports a canine form of the disease observed in a litter of Segugio Italiano dogs. Affected dogs, examined at 21 months old, presented with the core GAPO phenotype, short limbs, craniofacial abnormalities, alopecia, pseudoanodontia, and ocular abnormalities that included glaucoma. Whole genome sequence analysis of two affected puppies and the dam identified a nonsense variant c.505C > T in *ANTXR1* which introduces an early stop codon in exon 7, predicted to truncate the protein by 395 amino acids. Comparative quantification of *ANTXR1* gene expression in corneal, retinal and optic nerve tissues of a GAPO-affected dog were markedly reduced compared to control samples. Evaluation of protein expression through Western blot analysis using retinal and optic nerve tissue showed absence of ANTXR1 in the affected dog. Access to canine ocular tissues, rarely available in human GAPO patients, provides insight into tissue-specific effects of ANTXR1 loss. Our findings establish this canine disorder as a model of human GAPO syndrome and has facilitated the development of a DNA test that can identify carriers of the disease variant and prevent production of affected litters with this life limiting disease.

## Introduction

Canine skeletal dysplasias that include ocular abnormalities with a known genetic cause have been described in a number of dog breeds including the Labrador Retriever, Viszla, Dogo Argentino, Samoyed, Cane Corso and Northern Inuit [1–6]. In the Cane Corso, Samoyed, Labrador and Northern Inuit, skeletal dysplasias are syndromic, affecting additional tissues and organs, and all forms include ocular abnormalities. The Northern Inuit, Samoyed and Labrador skeletal dysplasia syndromes are known as oculo-skeletal dysplasia (OSD), caused by variants located in collagen type genes *COL9A2* and *COL9A3*. OSD-affected dogs of these breeds have short-limbed dwarfism and ocular abnormalities that include cataract, retinal detachment and vitreopathy [5,6]. The Cane Corso syndrome is termed Dental-Skeletal-Retinal-Anomaly (DSRA) and is characterised by disproportionate growth, brittle and translucent teeth, and progressive retinal degeneration [4]. The condition is caused by a splice site variant that causes the skipping of two exons within the *MIA3* gene.

This study was established to investigate the genetic cause of a novel syndromic skeletal dysplasia with ocular abnormalities observed in a litter of smooth haired Segugio Italiano puppies based in Italy. The Royal Kennel Club (UK) declassified the Segugio Italiano in 2020, as no new registrations had been requested since 2007 [7]. The breed is however registered with the Italian Kennel Club (ENCI) and registrations totalled 3204 hounds as of 2024 [8].

## Materials and methods

### Sample collection

Segugio Italiano (SI) tissue and DNA samples were submitted to the Canine Genetics Centre. DNA samples were submitted as buccal mucosal swabs or residual blood samples (from veterinary procedures), with owner consent (Ethical approval by the University of Cambridge Department of Veterinary Medicine Ethics and Welfare Committee No. CR863 2024, CR695, 2023 & CR496, 2021). DNA was extracted from buccal swabs using QIAamp DNA Blood Mini or Midi Kits (Qiagen, Manchester, UK). DNA was extracted from residual blood samples using a standard Nucleon protocol. This study was performed in accordance with the ARVO Statement for Use of Animals in Research. GAPO syndrome affected dogs (*n* = 3) were full siblings and diagnosed by a veterinary ophthalmologist. Control SI dogs (*n* = 25) were confirmed by a veterinary ophthalmologist as unaffected by GAPO syndrome. The eyes from an affected SI were collected post-mortem with owner consent. The dog was euthanised due to a severe decline in health as a consequence of the condition. One eye was placed in buffered formalin for histopathological evaluation and the other in RNAlater (Invitrogen). Control ocular tissue was collected from a 9.8 year old Golden Retriever who was euthanised owing to a terminal condition unrelated to GAPO syndrome. The tissue was provided with owner consent.

RNA was extracted from ocular tissues using the RNeasy Plus Mini Kit (Qiagen) according to the manufacturer’s instructions. Tissue was disrupted using Precellys 2 ml homogenizing tubes (CK14) (Bertin Health and Life Sciences) in a Precellys Evolution touch tissue homogenizer (Bertin Health and Life Sciences). For RNA extraction, a further step using QiAshredder columns (Qiagen) was also carried out. Complimentary DNA (cDNA) of *ANTXR1* was generated using the Omniscript RT Kit (Qiagen) in conjunction with RNAse Inhibitor (Applied Biosystems) and Oligo(dT) 12–18 Primer (Invitrogen).

### Ophthalmic investigation

An ophthalmic examination was performed on four SI siblings and their dam by a board-certified veterinary ophthalmologist at a specialty ophthalmology centre in Italy. Ophthalmic examination included: slit-lamp biomicroscopy (Kowa SL-17; Kowa Company Ltd., CA, USA), indirect ophthalmoscopy (Omega 600; Heine Optotechnik GmbH & Co., Gilching, Germany) with a 20D condenser lens, and direct ophthalmoscopy (PanOptic ophthalmoscope; Welch Allyn, NY, USA). Additionally, the following diagnostic tests were conducted: Schirmer tear test I (MSD Animal Health, Milton Keynes, UK), fluorescein staining (FluorOfta 1%; Fidia Farmaceutici S.p.A., Italy), applanation tonometry (Tono-Pen Vet; Medtronic, Jacksonville, FL, USA), central corneal pachymetry (PachPen; Accutome Inc., Phoenixville, PA, USA), and gonioscopy using a 17 mm Koeppe lens (Ocular Instruments, Bellevue, WA, USA).

### Histology

Histopathological examination of an enucleated globe removed post-mortem from a GAPO-affected SI was performed by CytoPath Limited, Herefordshire, UK.

### Exclusion of known canine inherited eye disease variants

Three GAPO-affected siblings were genotyped for previously reported canine inherited eye disease variants by massively parallel sequencing using a custom in-house designed amplicon array panel of PCR amplicons (published up to 2023). See Stanbury *et.al* (2024) for details [9].

### Whole-genome sequencing

Two GAPO-affected SI siblings and one SI control (unaffected dam of the affected siblings) were selected for whole genome sequencing (WGS). WGS was outsourced to Cancer Research UK, Cambridge Institute, University of Cambridge, where an Illumina 150 bp paired-end library was prepared and sequenced on the Illumina Novaseq 6000 platform, generating approximately 30X genome coverage. Read data were aligned to the CanFam4 UU Cfam GSD 1.0 reference genome using BWA-MEM v0.7 [10]. Base quality score recalibration, indel realignment and duplicate removal was performed using the Genome Analysis Toolkit (GATK) v4.2 according to GATK Best Practices recommendations [11,12]. SNP/INDEL discovery was performed using GATK HaplotypeCaller [12] and then loaded into a GenomicsDB. Joint variant calling was performed across 309 samples, including the three SI samples, and the resulting variant calls filtered using standard hard-filtering parameters. The filtered variants were annotated and functional effects predicted using SnpEff v5.1 [13] and visualised in the Integrative Genomics Viewer (IGV) software [14,15].

### WGS variant filtering

The first stage of variant filtering was performed against WGS of 306 dogs comprising 109 breeds and two cross breeds, via an in-house pipeline that prioritises variants based on the predicted effect on the protein. The 306 WGS consist of dogs with varying phenotypes excluding GAPO, therefore all 306 dogs acted as controls. The sire and dam of the SI GAPO-affected siblings were confirmed as unaffected by a veterinary ophthalmologist indicating a recessive mode of inheritance for the disease. Therefore a criterion of the variant filtering was that both cases had to be homozygous for an alternate allele and controls either heterozygous or homozygous for the reference allele. Variants retained after the first stage of filtering, with the highest predicted effect score and homozygous in both cases were then further filtered against a Variant Call Format (VCF) file containing 1987 WGS consisting of 1611 dogs (321 breeds), 309 village dogs, 63 wolves, and four coyotes curated by the Dog10K Consortium [16]. Genes containing potential candidate variants were cross-referenced with keywords (S1 Table) relating to GAPO syndrome using the NGS phenotyper software VarElect [17]. Pathogenicity of candidate variants was analysed using Polyphen-2 [18] and UMD-Predictor Pro [19]. UMD-Predictor Pro uses the human reference genome (GRCh38) to calculate pathogenicity of variants. Therefore, to ascertain if this *in-silico* tool was suitable for canine variants, human and canine proteins were aligned using Uniprot [20].

### Genotyping of WGS filtered variant by Sanger sequencing

Variants common in multiple breeds and/or predicted to be benign were excluded from further analyses. One variant remained after filtering as detailed above was homozygous in both GAPO-affected SI and heterozygous in the dam. The variant was a single nucleotide variant (SNV) located on chromosome 10 within exon 7 of the *ANTXR1* gene*.* Primers were designed to flank the variant using Primer3 (S2 Table) [21]. The variant was verified initially by Sanger sequencing of a further GAPO case (litter mate of probands) and two control SI (unaffected litter mate and unaffected maternal aunt of probands). PCR products were amplified using HotstarTaq DNA Polymerase (Qiagen). Cycling conditions were: 98^o^C for 10 mins; 35 cycles at 98^o^C for 30s; 57^o^C for 30s; 72^o^C for 30s; and 72^o^C for 5mins. Amplified products were Sanger sequenced in both directions at Source Bioscience, Cambridge, UK. Sequence traces were analysed using the Staden software package [22]. Further variant validation was carried out in a cohort of 22 unaffected SI.

### qRT-PCR

PrimeTime qPCR primers and probes were designed using the PrimerQuest Tool (IDT) and to bridge exon boundaries (the forward primer bridges exon 2–3 and exon 3–4 on the reverse) of the target gene *ANTXR1* and two housekeeping genes, *RPS5, RPS19* (S2 Table), selected based on Brinkhof *et.al* (2006) [23]. qRT-PCR assays were carried out using a StepOne Plus Real Time system (Applied Biosystems) in 20ul reaction volumes containing PrimeTime qPCR Assays (IDT) (FAM dye and ZEN quencher), and Luna Universal qPCR Master Mix (New England Biolabs). cDNA derived from corneal, retinal and optic nerve tissue of a GAPO-affected SI and an unaffected Labrador were analysed in triplicate using the Standard Curve quantification to ascertain reaction efficiency and then using the comparative C_T_ (ΔΔC_T_) method. Data were analysed using StepOne Software v.2.3.

### Western blot analysis

3 mg retinal and optic nerve tissue from a GAPO-affected SI and a control (GAPO-unaffected) dog were homogenised in radioimmunoprecipitation assay (RIPA) buffer (Cell Signalling Technology) and disrupted using a Precellys Evolution touch tissue homogenizer (Bertin Health and Life Sciences). Samples were spun at 16,000 xg for 10 minutes at 4^o^C and the soluble fraction collected. Denatured proteins were separated by SDS-PAGE in a NuPAGE Bis-Tris precast gel (Thermo Scientific). Protein transfer was carried out using Bio-Rad Trans-Blot Turbo Transfer RTA kit PVDF (Bio_Rad, #1704272) followed by blocking of the membrane in 5% Blotto and subsequent antibody treatments also in 5% Blotto. There are no ANTXR1 antibodies that have been validated in canines, therefore c37, an anti-rabbit monoclonal antibody was selected as the binding epitope region is identical in the dog to the human protein [24]. Membrane was incubated overnight with the rabbit monoclonal anti-TEM8/ANTXR1 antibody (c37) (Abcam, EPNCI-R173-37, 1:1000), followed by an HRP-linked goat anti-rabbit antibody (ThermoFisher, #31460, 1:20,000). The anti-TEM8/ANTXR1 antibody binding site is located in the von Willebrand factor Type A domain near the N-terminus of ANTXR1 (see supplementary data of Szot *et al.* 2018) [24]. After 30 minutes treatment with Restore Western Blot Stripping Buffer (ThermoFisher, #21059), the membrane was re-blocked and treated using mouse anti-β-actin (ThermoFisher, 15G5A11/E2, 1:5000), then HRP-linked donkey anti-mouse (SA1–100, ThermoFisher). Proteins were detected using SuperSignal West Pico Plus Chemiluminescent Substrate (Thermo Scientific) and visualised on a GeneGnome chemiluminescence imager (Syngene, GeneGnome XRQ).

## Results

### Clinical characterisation of GAPO cases

On general physical examination, subjects affected with the GAPO phenotype presented with skeletal dysmorphism, short limbs, pseudoanodontia, and diffuse dorsal alopecia (Fig 1).

**Fig 1:**
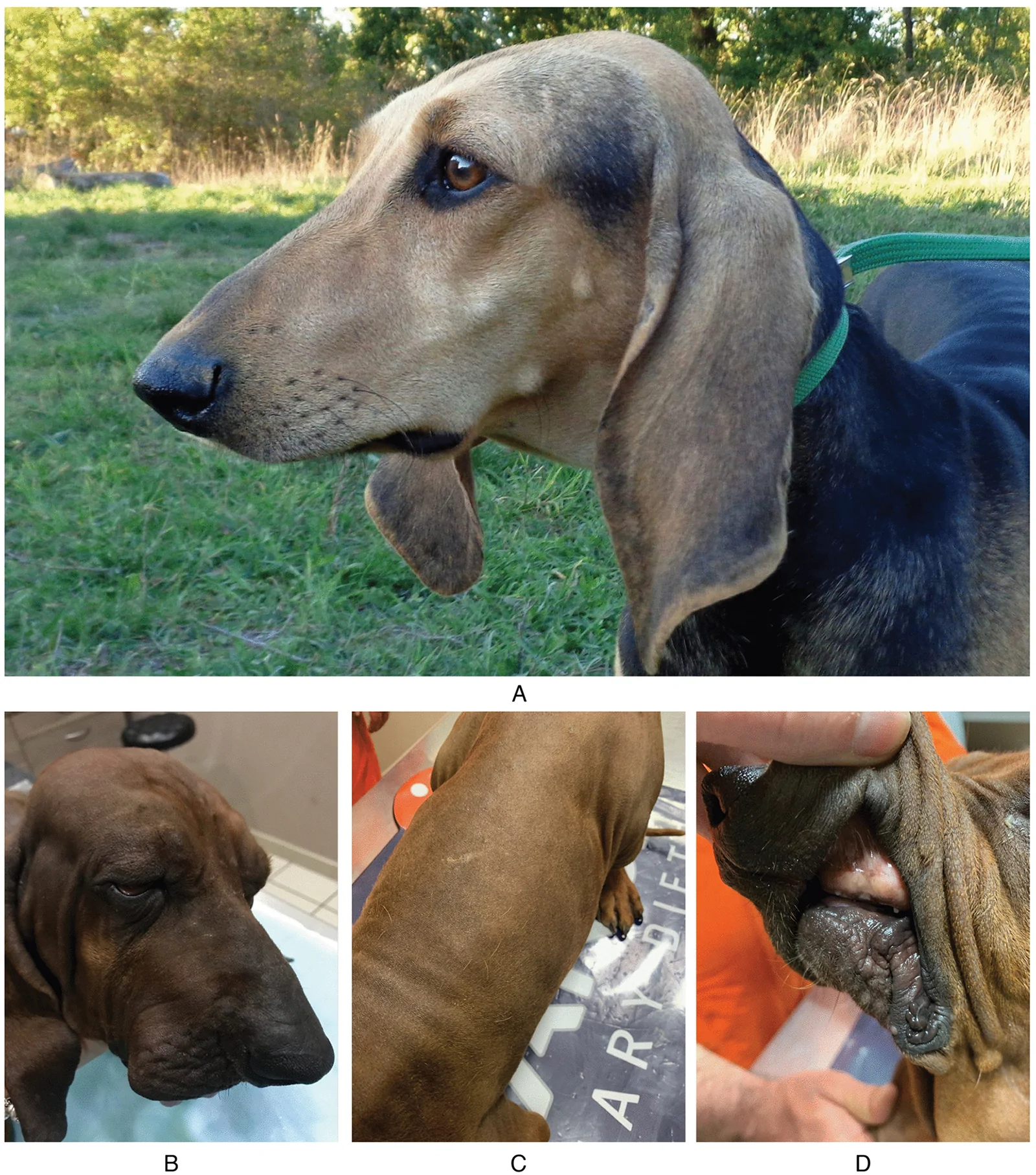
Photographs of a GAPO-unaffected and a GAPO-affected Segugio Italiano dog. **(A)** A GAPO-unaffected Segugio italiano dog. **(B) To (D)** – photos of a GAPO-affected Segugio Italiano dog. **(B)** Craniofacial anomalies: Skull dimorphism, short nose, micrognathia. **(C)** Dorsal alopecia. **(D)** Pseudoanodontia. Teeth have failed to erupt.

Ophthalmological evaluation revealed that the two male dogs had a positive menace response and direct and consensual pupillary light reflexes (PLR). In contrast, the affected female had a negative menace response and was observed bumping into objects during a maze test. The direct and consensual PLR were absent in this female due to nonresponsive mydriasis.

All affected subjects exhibited mild conjunctival hyperemia, mild to moderate scleritis, perilimbal corneal edema, small foci of intrastromal edema, and Haab’s striae (Fig 2). The anterior chamber was deep and clear. The iris showed nonresponsive mydriasis in one subject (the female) and was unremarkable in the others. Funduscopic examination revealed excavated optic nerve heads, an increased tapetal reflex, and retinal vessel tortuosity.

**Fig 2:**
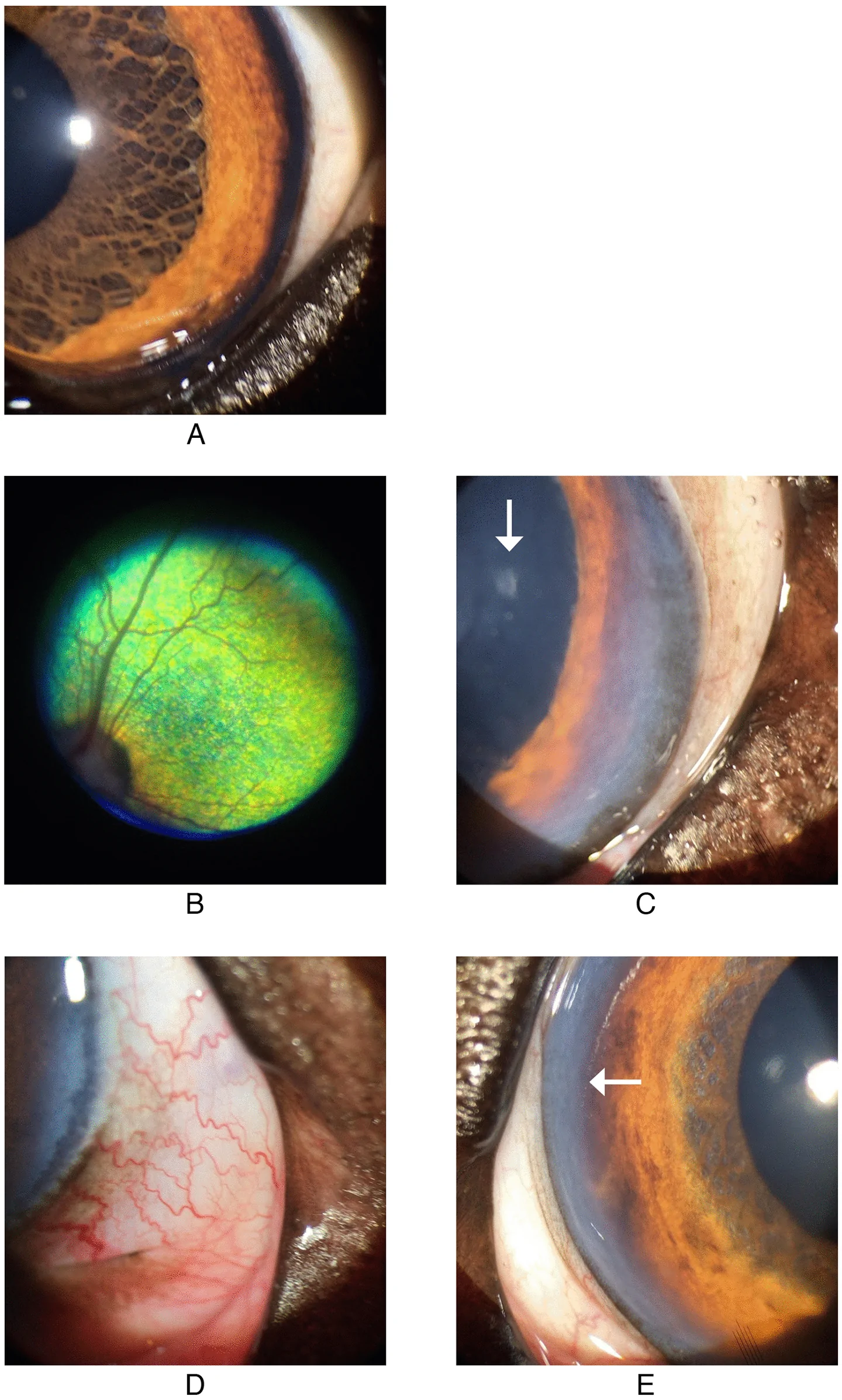
Ocular photographs of unaffected Dam and GAPO-affected Segugio Italiano dogs. **(A)** Photograph of left eye of unaffected Dam. **(B)** Left eye fundus photograph of Male #1 – Tapetal hypereflectivity. **(C)** Left eye of Male #1 – White arrow indicates small foci of intrastromal edema. **(D)** Left eye of Male #2 – Abnormal sclera-conjunctival vascularization. **(E)** Right eye of Male #1 – White arrow indicates perilimbal corneal edema.

Intraocular pressure (IOP) was elevated in the affected subjects, ranging from 40 to 67 mmHg (mean: 51 mmHg ± 9.5 SD) (Table 1). Gonioscopy revealed an open iridocorneal angle (ICA). The ICA was assessed by comparing the length of the pectinate ligament (PL) to the distance from the PL origin to the corneal endothelium at the transition zone. The pectinate ligament was classified as normal based on the European College of Veterinary Ophthalmologists (ECVO) manual, although rare fibrae latae and laminae were observed.

**Table 1:** Phenotypic characteristics of the Segugio Italiano dog litter and the unaffected dam.

	Dam	Male#1	Male #2	Male#3	Female
**Phenotype**	normal	affected	affected	normal	affected
**Skeletal dysmorphism**	absent	present	present	absent	present
**Pseudoanodontia**	absent	present	present	absent	present
**Alopecia**	absent	present	present	absent	present
**Menace Response**	+	+	+	+	-/+
**PLR**	+	+	+	+	Mydriasis
**Anterior Segment**	normal	Perilimbal edema/ Haab’s striae/ Scleritis	Perilimbal edema/ Scleritis	normal	Perilimbal edema / Haab’s striae/ Scleritis
**Gonioscopy**	normal	Open angle – fibrae latae < 10%	Not performed	normal	Open angle – laminae <25%
**IOP / mmHg** (Normal 10–25)	OD:18 OS:21	OD:56 OS:50	OD:40 OS:44	OD:18 OS:18	OD:67OS:48
**Pachymetry** (Normal 535–598)	OD:541 OS:548	OD:601OS:613	OD:659 OS:664	OD:558 OS:550	OD:648 OS:654
**Fundus**	Normal	Cupping ON retinal changes	Cupping ON retinal changes	Normal	Cupping ON retinal changes

Table legend: intraocular pressure (IOP); optic nerve (ON); right eye (OD); left eye (OS)

Central corneal pachymetry in affected dogs ranged from 601 to 664 microns (mean: 640 microns ± 26 SD) (Table 1).

Unaffected subjects were normal on both general physical and ophthalmic examinations. Their IOP ranged from 18 to 21 mmHg (mean: 19 mmHg ± 1.5 SD), and central corneal pachymetry ranged from 541 to 550 microns (mean: 550 microns ± 7 SD) (Table 1).

A follow-up of the two remaining affected SI dogs (Male#1 and Female) was carried out approximately 2 years after commencement of the study. Male#1 had been euthanized as his condition was reported to have declined. The female’s ophthalmological phenotype had progressed to total blindness with bilateral buphthalmos.

### Histology results

At the level of the drainage angle, the ciliary cleft and trabecular meshwork were open and there was no evidence of pectinate ligament abnormality (PLA). In the posterior segment, there was diffuse inner retinal atrophy with ganglion cell loss. The optic nerve head was degenerate and cupped. Within the cornea, there was a focal anterior stromal irregularity characterised by small clear clefts separating the collagen fibres and an accumulation of poorly defined/feathery to microvesicular vacuoles within the cytoplasm of the keratocytes. There is no macroscopic or histopathologic evidence of any other significant cause for glaucoma and overall findings are consistent with primary open angle glaucoma (POAG).

### WGS variant analysis

WGS of 306 dogs (differing health status and breed) acted as controls in the first stage of the variant filtering process. Analysis identified six variants homozygous in the GAPO-affected SI siblings and heterozygous in the dam. The six variants were exclusive to these three samples within the 306 WGS investigated initially. Three variants were subsequently eliminated after they were found to be common in multiple breeds within the Dog10K VCF [16]. A further two variants located in genes *WIPF1* and *DOK7* were then eliminated on the basis that VarElect [25] denoted their association with the phenotype to be ‘Indirect’ and Polyphen-2 [18] predicted both to have a ‘benign’ effect on the protein with pathogenicity scores of 0.001 and 0.004 respectively. The remaining variant was identified as nonsense and located in *ANTXR1.* The human and canine ANTXR1 alignment shows that the protein is highly conserved between these two species (S1 Appendix) and therefore AMD-Predictor Pro was used to calculate pathogenicity. The c.505 C > T single nucleotide variant (SNV) was predicted to be highly pathogenic with a score of 100 [19]. The variant would be classified as pathogenic (PVS1) according to variant classification guidelines [26].

### *ANTXR1* variant validation

The *ANTXR1* c.505C > T (p.R169*) variant located at g.chr10:69183369 (Fig 3) was genotyped by Sanger sequencing in the maternal aunt and two further littermates (one affected and one unaffected) of the probands, and 22 GAPO-unaffected SI (Table 2).

**Table 2:** Combined results of *ANTXR1* nonsense c.505C > T variant by whole genome sequencing and genotyping by Sanger sequencing.

Disease Status	Homozygous Alternate (T/T) +/+	Heterozygous (C/T) +/-	Homozygous Wild Type (C/C)-/-	Total
**GAPO Cases**	3	0	0	**3**
**Controls**	0	2	23	**25**

**Fig 3:**
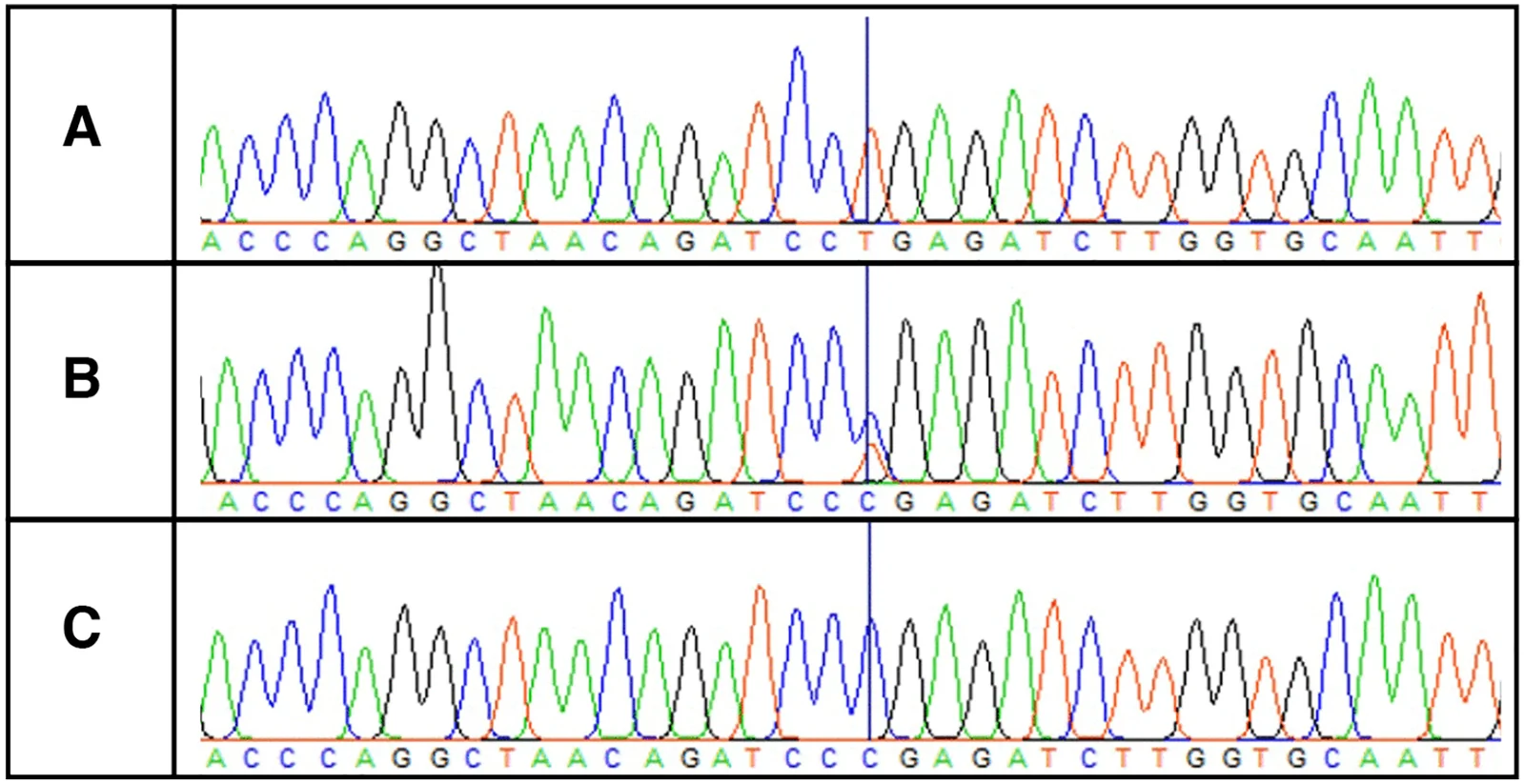
Nonsense variant identified in *ANTXR1.* Illustration of the *ANTXR1* c.505C > T nonsense variant located at chr10:69183369 (UU_Cfam_GSD_1.0, Canfam4) Electropherogram Sanger sequencing reads of (**A**) GAPO-affected SI (T/T), (**B**) unaffected littermate (T/C) and (**C**) unaffected maternal aunt (C/C).

### Comparative gene expression of *ANTXR1*

Comparative mRNA gene expression analysis of *ANTXR1* normalised to RPS19 and RPS5 in retinal, corneal and optic nerve tissue showed decreased levels in the GAPO-affected SI compared to a GAPO-unaffected control (Fig 4). Relative expression in the GAPO-affected SI cornea was 0.03. Control retinal *ANTXR1* expression was 0.53 compared to 0.09 in the GAPO-affected SI. The highest expression was observed in optic nerve tissue in the control with a level of 3.28 and 0.10 in the GAPO-affected SI.

**Fig 4:**
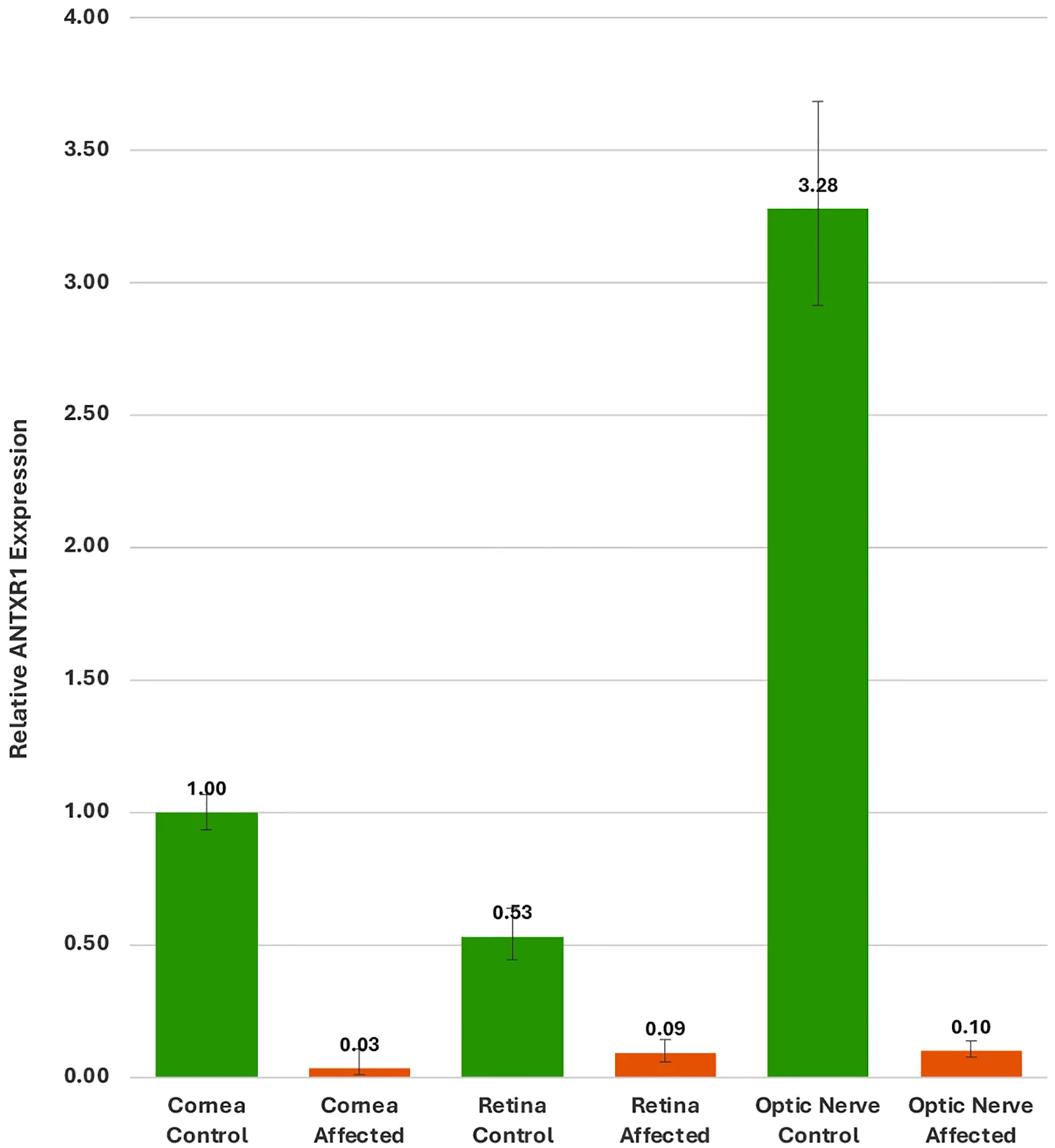
Comparative quantification of *ANTXR1* mRNA. Comparative quantification of mRNA in corneal, retinal and optic nerve tissue from a GAPO-unaffected (control) dog and a GAPO-affected SI using the comparative ΔΔCt method. *ANTXR1* mRNA was normalised to Ribosomal Proteins S19 and S5 (*RPS19* and *RPS5*) and using Control Cornea as a calibrator sample. Error bars represent ± standard deviation of assays run in triplicate.

### Western blot analysis

Western blot analysis showed absence of ANTXR1 in the GAPO-affected SI ocular tissues (Fig 5) and presence in control tissues with an approximate size of 80-90kDa (S1 Fig). Control blot of ß-Actin showed presence of protein approximately 45kDa in size (S2 Fig) in cases and controls and in both ocular tissues.

**Fig 5:**
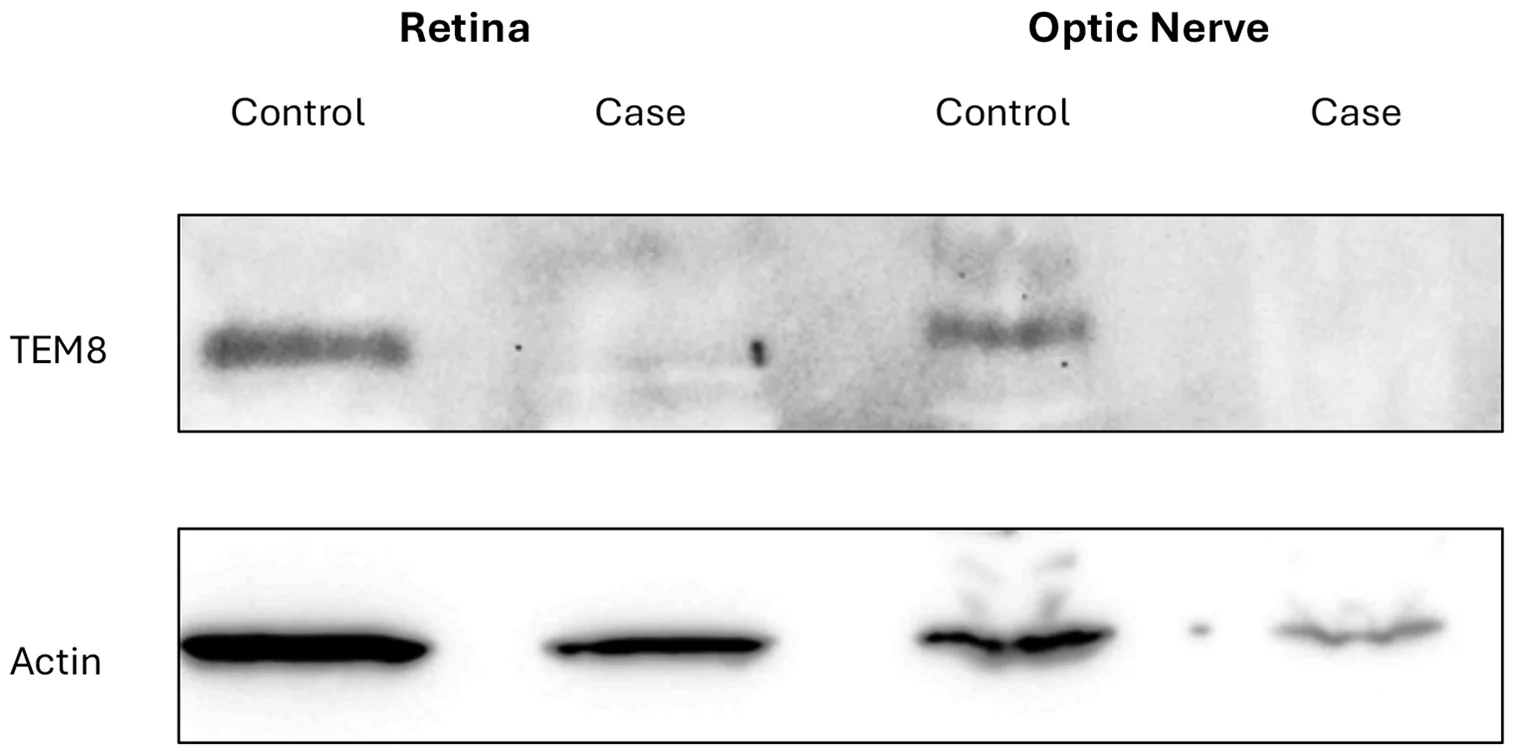
Western Blot analysis of ANTXR1 (TEM8) protein in two ocular tissues. The TEM8 (ANTXR1) results in retinal and corneal tissue in a GAPO-affected and control dog are shown in the top box. Results of ß-Actin protein blotting are shown in the bottom box.

## Discussion

In this study we describe a novel syndromic disease observed in the Segugio Italiano dog breed. We used whole genome sequence analysis of two affected SI and the unaffected dam to identify a causal variant located in the Anthrax Toxin Receptor 1 gene (*ANTXR1*). The affected SI displayed a phenotype that included short limbed dwarfism, craniofacial abnormalities, dorsal alopecia, pseudoanodontia and ocular abnormalities that included glaucoma. Whilst these disease manifestations have a degree of overlap with canine syndromic diseases such as dental-skeletal-retinal-anomaly [4] and oculoskeletal dysplasia [5,6,27], or Marfan syndrome in humans [28], none of these conditions report alopecia and pseudoanodontia as part of the phenotype. Furthermore, the ocular abnormalities in the aforementioned conditions were not consistent with the clinical signs observed in the affected SI dogs. The identification of the nonsense *ANTXR1* variant allowed for both a clinical and molecular diagnosis for the affected SI which we propose is a canine form of GAPO syndrome.

GAPO syndrome is a human condition characterised by four primary features: **g**rowth retardation, **a**lopecia, **p**seudoanodontia and **o**cular atrophy, all of which were observed in the affected SI. GAPO syndrome has to date, only been reported in humans and is caused by pathogenic variants in the Anthrax Toxin Receptor 1 gene, with a recessive mode of inheritance [29–34]. The condition is extremely rare in humans with just 105 cases reported, according to a recent literature review [35]. Whilst we had a modest number of control SI available for this study (Table 2), we speculate that the condition is also rare in the dog. As far as the authors are aware this is the first report of the phenotype in this species, and furthermore the c.505C > T *ANTXR1* variant identified was exclusive not only to the SI breed but also to the probands’ family (after filtering against the Dog10K and in-house VCF files).

ANTXR1 is a transmembrane glycoprotein comprised of an extracellular region containing a von Willebrand factor type A domain (vWA), a transmembrane domain and a cytoplasmic tail which binds actin [29,36,37]. There are 5 proposed *ANTXR1* isoforms in the human, two of which (V3 and V5) are presumed to be secreted as they lack both the transmembrane and cytoplasmic regions [29,38]. *ANTXR1* is reported to be widely expressed in human tissues [39] and according to data collated by the DoGA Consortium [40], is expressed across all canine organ systems, with the highest levels reported in the cardiovascular system, and the second highest in the eye. Owing to the differentially spliced variants of the protein, *ANTXR1* expression is thought to have tissue specific functionality, and mouse studies have reported higher levels during embryonic development than observed in adult tissue [24,37,38,41].

The c.505C > T variant identified in the GAPO-affected SI is located at p.169 of the ANTXR1 protein, which is located in exon 7 (all transcripts) within the vWA domain. This region is present within all isoforms of both the human and canine proteins and is within a highly conserved region across mammals (S2 Appendix). Comparative analysis of corneal, retinal and optic nerve *ANTXR1* mRNA expression in a GAPO-affected SI and a control dog (aged 9.8yrs) was carried out to ascertain if the early termination codon introduced by the c.505C > T variant triggered nonsense-mediated mRNA decay (NMD) (Fig 4). The NMD pathway acts as a quality control mechanism to avoid production of truncated and therefore possibly pathogenic proteins, however this pathway is not ubiquitous across all cell and tissue types [42]. Our results (Fig 4) demonstrate that optic nerve has the highest level of expression in the ocular control tissues. This is consistent with human data, where optic nerve *ANTXR1* mRNA expression is approximately x8 greater than in the retina and also with canine data which is approximately x5.5 greater [40,43]. *ANTXR1* mRNA in GAPO-affected SI retinal tissue was almost 6 times lower than that observed in the control and almost negligible in the corneal and optic nerve tissues. This suggests the aberrant transcript(s) in the GAPO-affected SI undergo partial degradation via the NMD pathway. If developmental expression levels in the dog mirror those of the mouse, and decrease with age, as the difference we observed in expression between the affected SI and the control dog could be an underestimate as the control dog was approximately 10 years old and the GAPO SI case was approximately 2 years old. Similar findings were reported by Stránecký et al. (2013), who performed relative expression analysis of *ANTXR1* in human skin fibroblasts. This group was also instrumental in identifying *ANTXR1* as the causative gene for GAPO syndrome [27]. Strikingly, their study reported one human GAPO-affected individual that was homozygous for the identical variant (c.505C > T; p.R169*) that we have identified in the SI-affected dogs described here [27]. In addition to negligible gene expression levels, the p.R169* variant in the GAPO-affected human patient resulted in a loss of ANTXR1 production in skin fibroblasts [29]. We obtained a commensurate result, demonstrating that ANTXR1 was absent from retinal and optic nerve tissues in the affected SI (Fig 5).

In addition to gene and protein expression analysis, Stránecký and co-authors assayed functional effects that the nonsense variant had on actin, microtubules and intermediate filaments in cultured skin fibroblasts. Microtubule and intermediate filament architecture appeared unchanged, however actin cytoskeletal microfilaments underwent a complete reorganisation compared to controls. This strongly suggests that ANTXR1 is vital for correct actin assembly.

The GAPO-affected SI presented with abnormalities in multiple organ systems; integumentary, nervous and skeletal (Figs 1 and 2). GAPO-affected humans have comparable abnormalities within the same organ systems, but abnormalities of the cardiovascular, respiratory and reproductive systems have also been reported [35]. Whilst the exact physiological functions of *ANTXR1* have yet to be defined [37], studies of the protein within varying tissues have suggested roles that include cellular mechanosensing [36], cellular senescence [37] and extracellular matrix homeostasis [29,39,41]. A major contributor to the pathogenesis of GAPO syndrome is attributed to a malfunction of the latter role, whereby there is an abnormal accumulation of extracellular matrix components and generalised interstitial fibrosis within tissues. The disease is progressive as a consequence of continual extracellular matrix deposition, which ultimately leads to a reduced life expectancy of affected individuals [29,36,37,44]. Follow up of the two surviving GAPO-affected SI found that the female has progressed to complete blindness with buphthalmos and the male was euthanised due to a severe decline in health.

The ophthalmic feature of GAPO syndrome is termed ‘optic atrophy’, however, ocular abnormalities in the human vary between individuals, and therefore ‘optic manifestations’ has been suggested by some to better reflect the heterogeneity of this aspect of the condition [31,45,46]. Optic atrophy is reported in 46% of human GAPO patients [35] however, it is possible that the real figure could be higher. A diagnosis of optic atrophy was confirmed in all patients who underwent fundoscopy, and therefore it appears that the inclusion of this phenotype is dependent on a comprehensive ophthalmological examination being carried out [35]. Ocular abnormalities observed in the GAPO-affected SI after clinical and histopathological examination were located in the retina, cornea and fundus. All GAPO-affected SI showed retinal vessel tortuosity, a finding also observed in GAPO-affected humans [29,47,48]. Loss of *ANTXR1* function in endothelial cells has been linked to abnormal angiogenesis in the human and chicken, with the latter developing irregular vessel patterning and density in the chorioallantoic membrane [41,49]. Retinal vascular tortuosity can occur as a natural effect of ageing, but can also occur due to conditions such as hypertension, diabetes mellitus or underlying genetic defects [50]. It has also been hypothesised to be associated with glaucoma progression [51]. The relationship between glaucoma and the vascular branching is thought to be via reduced blood flow which induces retinal ganglion cell death [51]. As ganglion cells progressively die, optic nerve cupping occurs which is a known glaucoma risk factor [52]. All GAPO-affected SI showed optic nerve cupping and were diagnosed with glaucoma, and histopathology revealed widespread retinal atrophy with ganglion cell loss.

Canine glaucoma is classed as primary in the absence of any precursory ocular conditions and then further subdivided into either open-angle or closed-angle glaucoma according to the architecture of the iridocorneal angle (ICA) [53]. The two GAPO-affected SI who underwent gonioscopy had an open ICA and normal pectinate ligament (according to ECVO classification of <50% *fibrae latae*) [54], a finding confirmed by histopathology in the third affected SI. Therefore, a diagnosis of open-angle glaucoma was made in the SI dogs. Glaucoma is an inconsistent feature of GAPO syndrome in humans which has led some to speculate if additional disorders are involved [31–33]. Many clinical reports of human GAPO patients pre-date the identification of the genetic cause. Therefore, it is also possible that it is the nature of the *ANTXR1* variant itself that contributes to the development of glaucoma. To date 20 variants have been identified as causal for GAPO syndrome and the majority of these studies, when it has been tested for, have identified glaucoma as a clinical sign [29,32–35,55–61]. However, a molecular and clinical study of three GAPO-affected siblings found that only the two youngest children had congenital glaucoma [32]. They all shared a homozygous missense *ANTXR1* variant (c.572T > C, p.Ile191Thr) which did not apparently explain the glaucoma diagnosis. Further analysis identified an additional, heterozygous variant in the two glaucoma-affected sisters located in *TGFB1*. This gene is compelling as it has been implicated in glaucoma pathogenesis in prior studies [62,63]. However, it should also be noted that the study of the three siblings employed a whole exome sequencing approach to identify the missense *ANTXR1* variant and therefore, any intronic variants potentially involved in splicing will have not been identified.

Histopathology of the affected SI identified a focal anterior corneal stromal anomaly and an accumulation of an unknown substance within the cytoplasm of keratocytes. The accumulated material was postulated to consist of either lipids or a possible aggregation of glycoprotein/mucin. In light of the GAPO syndrome diagnosis, it is feasible that this substance is an accumulation of extracellular matrix components occurring within the corneal tissue. Furthermore, collagen type I is the most abundant collagen type within the corneal stroma, and a binding target of *ANTXR1* [36,64]. Ophthalmological examination revealed perlimbal corneal edema in all three GAPO-affected SI (Fig 2). Pachymetry calculations of the SI family (Table 1) showed a 16.6% mean increase in corneal thickness in the affected dogs compared to the dam and unaffected sibling, an occurrence that has also observed in GAPO-affected humans [32]. Variability of corneal thickness can occur due to factors such as age and sex, but also inflammation and glaucoma [65–67]. The corneal phenomena in the SI however, were reported to have occurred in the absence of keratitis. The increase may instead be as a consequence of extracellular matrix accumulation within this tissue as a consequence of negligible *ANTXR1* expression and furthermore that the increased corneal thickness is a contributing factor to the open-angle type glaucoma in the affected SI [66].

In conclusion we have identified a nonsense variant in the Segugio Italiano dog breed that causes a phenotype identical to GAPO syndrome in humans. Consistent with the human condition, the canine phenotype is progressive, as demonstrated by the clinical deterioration of the two dogs followed up after initial ophthalmology consultations. This study advances understanding of this rare disease by presenting novel findings using tissue types that are not readily accessible in human GAPO patients, and expands on the work of Stránecký and colleagues who identified an identical nonsense *ANTXR1* variant in a human GAPO patient [29]. These results further highlight the value of the dog as a model for human disease, and reinforce the broader relevance of naturally occurring canine genetic disorders to comparative biomedical research [9,27,68].

## Supporting information

S1 Table Phenotypic keywords used for Varelect. (DOCX)

S2 Table Primers used for sanger sequencing and qPCR. (DOTX)

S1 Appendix Alignment of human and canine ANTXR1 protein. Reprinted from Uniprot under a CC BY Licence 4.0 with permission from the Uniprot Consortium, copyright 2022.
(TIF)

S2 Appendix NCBI multiple sequence alignment showing the highly conserved region of ANTXR1 in mammals where the early termination codon is introduced (shown in a black box). Reprinted from National Library of Medicine with permission from The National Library of Medicine and the U.S. Government.
(TIF)

S1 Fig TEM8/ANTXR1 western blot image results. (TIF)

S2 Fig β-actin western blot image results. (TIF)
