# Supplementary material for: Genetic characterization of a canine model of human GAPO syndrome caused by a nonsense variant in ANTXR1
Source: PLoS One. 2026 Oct 26;21(10):e0359081. doi: 10.1371/journal.pone.0359081 (PMC13704829; doi:10.1371/journal.pone.0359081)
Supplement: Supplementary file 1 [file pone.0359081.s001.docx]

**S1 Table. Phenotypic keywords used for Varelect**

Optic nerve cupping

Skeletal abnormality

Glaucoma

Mandible abnormalities

Dental agenesis

Craniofacial abnormality

Hair abnormality

Short limb

Craniomaxillofacial anomaly

Dental abnormalities

Alopecia

Pseudoanodontia
