# Supplementary material for: Genetic characterization of a canine model of human GAPO syndrome caused by a nonsense variant in ANTXR1
Source: PLoS One. 2026 Oct 26;21(10):e0359081. doi: 10.1371/journal.pone.0359081 (PMC13704829; doi:10.1371/journal.pone.0359081)
Supplement: Supplementary file 2 [file pone.0359081.s002.dotx]

| **Primers used with gDNA for Sanger Sequencing** | | |
| --- | --- | --- |
| **Name** | **Sequence** | **Amplicon Size** |
| ANTXR1_F | CATCGGGTCCCTCATACATCG | 443bp |
| ANTXR1_R | CATTGGCTTGGCTTCATGTCC |  |

**S2 Table. Primers used for Sanger Sequencing and qPCR**

| **Primers used with gDNA for Sanger Sequencing** | | |
| --- | --- | --- |
| **Name** | **Sequence** | **Amplicon Size** |
| ANTXR1_F | CATCGGGTCCCTCATACATCG | 443bp |
| ANTXR1_R | CATTGGCTTGGCTTCATGTCC |  |

| **PrimeTime™ qPCR Probes – Dye = FAM and Quencher = ZEN** | | |
| --- | --- | --- |
| **ANTXR1** | | |
| Forward | TCCTGGACAAATCAGGAAGTG | Exon 2-3  Bridges exon 3-4  94bp |
| Reverse | CTTAGCTGTGGGCTGATGAA |  |
| Probe | TGCTGCACCACTGGAATGAAATCT |  |
| **RPS19** | | |
| Forward | GGAAGCTGAAAGTCCCTGAA | 107bp |
| Reverse | TGCTGTGGAAGCAGCTC |  |
| Probe | CCAGTTCTCATCGTAGGGAGCAAGC |  |
| **RPS5** | | |
| Forward | CGAGATCATCCATCTGCTCAC | 108bp |
| Reverse | GTTCCAGCTCGCCCAAT |  |
| Probe | TGCAGGTCCTGGTGAATGCCATTA |  |
